# Long-term Follow-up for Multidrug-resistant Tuberculosis

**DOI:** 10.3201/eid1204.041256

**Published:** 2006-04

**Authors:** Sonya S. Shin, Jennifer J. Furin, Felix Alcántara, Jaime Bayona, Epifanio Sánchez, Carole D. Mitnick

**Affiliations:** * Brigham and Women's Hospital, Boston, Massachusetts, USA;; † Socios en Salud, Lima, Peru;; ‡ Peruvian Ministry of Health, Lima, Peru;; § Harvard Medical School, Boston, Massachusetts, USA

**Keywords:** Tuberculosis, follow-up, outcomes, multidrug resistance, Peru, employment, relapse, dispatch

## Abstract

Patients treated in Peru for multidrug-resistant tuberculosis (MDR-TB) were followed-up for a median of 67 months. Among 86 patients considered cured after completion of treatment, 97% remain healthy; 1 patient relapsed. Employment increased from 34% before treatment to 71%. We observed favorable long-term outcomes among MDR-TB patients.

Increasing awareness of the rising global rates of multidrug-resistant tuberculosis (MDR-TB) has led to a concerted international effort to confront this disease, particularly in countries with a high incidence of TB (*1**–**3*). Nonetheless, despite cure rates >80% in some programs, MDR-TB patients tend to have chronic disease and require prolonged therapy.

Little is known about the long-term follow-up of patients treated for MDR-TB, including rates of relapse and chronic disability among cured persons. Among patients treated for pansusceptible TB, chronic disability caused by pulmonary sequelae and malnutrition can be substantial (*4*). Given the prolonged nature of MDR-TB, one might expect higher rates of chronic disability among patients with drug-resistant TB compared with those with pansusceptible TB. To explore these questions, we conducted long-term follow-up, defined as follow-up for a minimum of 4 years after treatment was initiated, of MDR-TB patients who received individualized therapy in Lima, Peru (*1*).

## The Study

We performed a retrospective study among all patients who initiated individualized, community-based MDR-TB therapy from August 1, 1996, to March 1, 2000. The details of patient identification, enrollment, and treatment are described elsewhere (*5*). Patients were resistant to a median of 5 drugs (range 2–9). Regimens generally included at least 5 drugs to which the infecting isolate was susceptible, and treatment duration was 18–24 months. Routine follow-up after completion of MDR-TB therapy included 1) routine smear microscopy and culture on sputum samples 1 month after completion of treatment and then every 6 months for 1 year; 2) subsequent smear microscopy, culture, and clinical evaluation by a TB physician for any episode of potential TB symptoms, e.g., a respiratory illness lasting >14 days, hemoptysis, or weight loss of unclear cause; and 3) continued contact with Socios en Salud (the community-based organization working with the Ministry of Health on this MDR-TB treatment project) through the network of health promoters, patient group therapy sessions, and a social assistance program. Thus, loss to follow-up or undocumented medical attention for respiratory illness is rare.

When reporting cohort outcomes, the MDR-TB working group recommends follow-up for 2 years from the time of treatment initiation when reporting cohort outcomes (*6*); however no international definition of long-term follow-up for MDR-TB cohorts exists. Therefore, we defined long-term follow-up as twice the duration set forth by the MDR-TB working group.

We conducted a chart review to determine TB-related symptoms and employment status of persons recorded at baseline by the intake physician before they received MDR-TB therapy. In addition to data obtained through routine patient follow-up as per program norms described above, Socios en Salud staff involved in the social assistance program, members of group therapy sessions, and health promoters were interviewed to obtain additional follow-up information about the patients, including income, employment status, and household information. A community health worker conducted home visits to interview all patients; patients were questioned about current symptoms as well as their socioeconomic status. The study was reviewed and approved by the institutional review board at Harvard Medical School; local institutional review was not required.

Among 120 persons enrolled in this study, 23 patients died during treatment, and 1 person remained in culture-negative treatment at the time of analysis. Two patients (both of whom had defaulted from treatment) were lost to follow-up.

Data are reported on the remaining 96 (80%) patients who were alive at the time of stopping MDR-TB therapy. Patients were followed for a median (95% confidence interval) of 67 (47–88) months after initiation of treatment and a median of 46 (3–84) months after completion of treatment. As summarized in the Table, 86 (72%) patients were considered cured, 9 (8%) defaulted from treatment, and 1 (1%) had failed treatment.

**Table:** Status of 96 multidrug-resistant tuberculosis patients after a median of 67 months of follow-up

Current status	Status at time of stopping treatment				
	Cured	Abandoned, culture positive	Abandoned, culture negative	Failed	Total
Alive, culture negative	83	0	2	0	85
Alive, culture positive	0	0	0	0	0
Died, culture positive	1	3	1	1	6
Died, culture negative	2	0	1	0	3
Unknown	0	0	2	0	2
Total	86	3	6	1	96

Among those who were considered cured at the time of treatment completion, 83 (97%) are currently healthy. One patient relapsed 1 month after completion of treatment; this patient refused retreatment and subsequently died of TB. Two other cured (culture-negative) patients later died (1 of a narcotic overdose and the other of respiratory insufficiency).

Of the 9 patients who defaulted, 3 were culture-positive at the time they abandoned treatment. Among the 9 defaulters, 5 died (4 from TB and 1 by suspected suicide), 2 are currently culture negative, and 2 were lost to follow-up since the time of treatment default. One patient was considered a treatment failure and, despite further retreatment regimens, subsequently died of TB.

Among 96 patients who were alive at treatment completion, 85 (89%) currently remain healthy. Thus, among the entire cohort of 120 patients enrolled during the study period, favorable long-term outcome was observed among 71%. Four patients experienced long-term sequelae: hemoptysis caused by aspergilloma necessitating pulmonary resection (1 patient), bronchiectasis and recurrent respiratory infections (2 patients), and bronchopleural fistula after pneumonectomy (1 patient).

In addition to medical care, Socios en Salud provides social assistance with financial support to resume work and pursue studies. Of the 96 patients, 21 patients (22%) received financial aid to pursue work or study, and 13 (14%) have had children since they were cured. Employment improved from 34% before therapy to 71% after therapy. None of the persons employed before starting MDR-TB treatment have lost their jobs because of work disruption caused by their TB therapy.

## Conclusions

Although MDR-TB presents a major challenge to TB control, effective treatment can result in cure. Long-term follow-up is important for understanding the long-term efficacy of treatment and the overall impact of this disease on patients' physical and socioeconomic well-being.

This study has several interesting findings. First, unlike previous reports of high death rates associated with MDR-TB, most of our patients met the definition of cure upon completion of treatment. Second, few patients who were cured at treatment completion had long-term sequelae or relapse. Third, most patients were able to resume work or studies and participate in family roles as parents and caretakers. Finally, the default rate was low (7.5%).

We recognize the limitations of this study. First, the cohort was small, and longer follow-up would be useful in determining if these indicators of physical and social recovery are sustained. Second, several patients were lost to follow-up; thus, the outcome of these patients is still not well characterized. Finally, these results may not be applicable to other situations, where the socioeconomic situation determines, in large part, the ability of a patient to resume work and studies.

With access to laboratory results to guide individualized therapy for persons with MDR-TB in the context of strong community-based support, the outcomes observed in Peru are encouraging. These outcomes favor the implementation of similar MDR-TB treatment programs globally.
